# COVID-19 Pneumomediastinum: Possible Role of Transesophageal Echo in Bedside Percutaneous Bicaval Double-Lumen ECMO Cannulation in Children. A Case Report

**DOI:** 10.3389/fped.2021.740853

**Published:** 2021-10-27

**Authors:** Andrea Moscatelli, Stefano Pezzato, Silvia Buratti, Elisabetta Lampugnani, Alberto Di Mascio, Maria Beatrice Damasio, Roberta Caorsi, Marco Gattorno, Elio Castagnola

**Affiliations:** ^1^Neonatal and Pediatric Intensive Care Unit, IRCCS Istituto Giannina Gaslini, Genoa, Italy; ^2^Radiology Unit, IRCCS Istituto Giannina Gaslini, Genoa, Italy; ^3^Rheumatology Unit, IRCCS Istituto Giannina Gaslini, Genoa, Italy; ^4^Infectious Disease Unit, IRCCS Istituto Giannina Gaslini, Genoa, Italy

**Keywords:** COVID-19, extracorporeal membrane oxygenation, percutaneous bicaval double-lumen ECMO cannulation, transesophageal echo, air leak syndrome

## Abstract

COVID-19 is generally uneventful in children. Only 8% of severe acute respiratory distress syndrome corona virus 2 pediatric patients require intensive care; of these, 1% may need extracorporeal membrane oxygenation. Preexisting medical conditions are an independent risk factor for pediatric intensive care unit admission. We describe the case of an 11-year-old girl with adenosine deaminase 2 deficiency who presented severe COVID-19 acute respiratory distress syndrome, complicated by a massive air leak syndrome. The respiratory failure, refractory to conventional support, required veno-venous extracorporeal membrane oxygenation. To prevent viral diffusion, bicaval double-lumen cannulation was performed percutaneously at the bedside under exclusive echo guidance. Because of pneumomediastinum, pneumothorax, and subcutaneous emphysema, ultrasound visualization of the heart was possible only with transesophageal echo. To our knowledge, this is the first description of a transesophageal echo guided bedside percutaneous bicaval double-lumen extracorporeal membrane oxygenation cannulation in a pediatric patient. Pitfalls of the technique are highlighted.

## Introduction

Coronavirus disease 2019 (COVID-19) presents generally an uncomplicated clinical course in children. According to recent data, 8% of severe acute respiratory syndrome coronavirus 2 (SARS-CoV-2)-infected pediatric patients need pediatric intensive care unit (PICU) admission and 4% undergo mechanical ventilation (MV), while <1% are assisted on extracorporeal membrane oxygenation (ECMO). Preexisting medical conditions like adenosine deaminase 2 deficiency (DADA2) are an independent risk factor for PICU admission. ECMO can be required for respiratory and/or circulatory support (1). Air leak syndrome (ALS), characterized by pneumothorax, pneumomediastinum, and interstitial and subcutaneous emphysema, may complicate SARS-CoV-2 pneumonia (2). In such cases, veno-venous ECMO should be the preferred modality for respiratory support, often representing an effective bridge to recovery. Bicaval double-lumen (BCDL) cannulation might be highly challenging because severe ALS can compromise the acoustic window needed for transthoracic echo (TTE) guidance at the bedside (3). For several reasons, bedside ECMO cannulation represents an invaluable option in the context of the COVID-19 pandemic. First, mobilization of the patient to an angiography facility is not required, containing the potential spread of the virus. Second, a limited number of personnel is exposed to the risk of infection since cannulation can be performed by three healthcare professionals: cannulating intensivist, assistant, and one intensivist performing the echo. Third, it is possible to optimize resource utilization in intensive care units overwhelmed by the burden of patients needing intensive care. This case report shows the feasibility of echo-guided bedside percutaneous BCDL cannulation in children when the absence of a transthoracic window requires transesophageal echo (TEE) control (3). In accordance with the European Union GDPR, informed consent has been asked from parents for the treatment of patient's data and images, including for scientific purposes.

## Case Report

**Figure 1:**
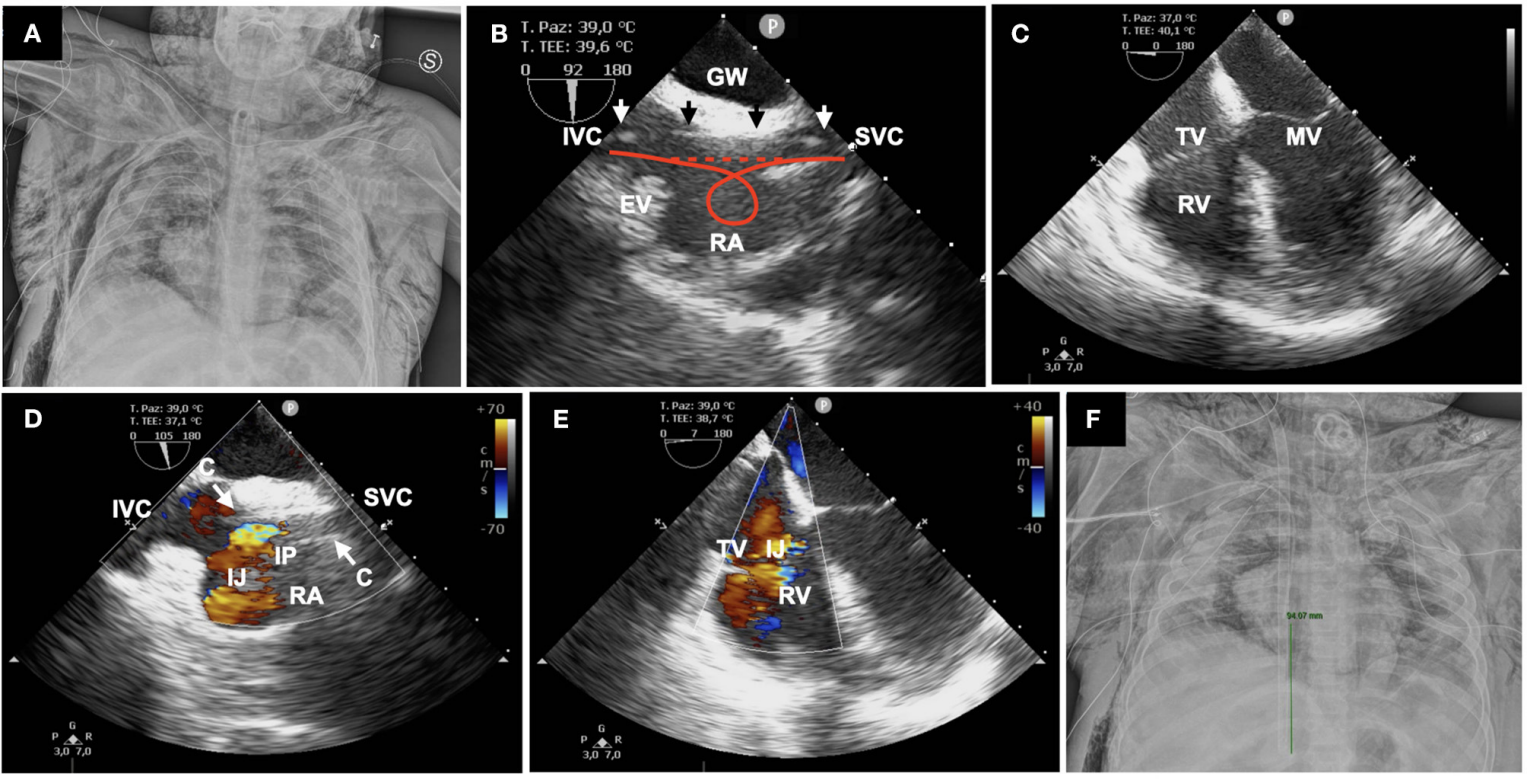
**(A)** Massive air leak syndrome with pneumomediastinum, bilateral pneumothorax, and subcutaneous emphysema. **(B)** GW (arrows) displayed straight from the SVC to IVC; the red lines show how a discontinuous hyperechoic image can help in ruling out looping; right atrium (RA), Eustachian valve (EV). **(C)** Absence of looping or GW malposition in the right ventricle (RV) is demonstrated in four-chamber MEV; tricuspid valve (TV), mitral valve (MV). **(D)** Mid-esophageal bicaval view showing the correct position of the cannula (C) with the infusion port (IP) in the middle of the right atrium; infusion jet (IJ). **(E)** Four-chamber MEV confirming the direction of the IJ toward the tricuspid valve. **(F)** Chest X-ray demonstrating the correct position of the cannula; the green line measures the tip-to-IP distance (9.4 cm).

An 11-year-old girl, 33-kg ideal body weight, with suspected DADA2 was referred to our PICU because of SARS-CoV-2 evolved to acute respiratory distress syndrome (ARDS). A complete deficiency at enzymatic testing confirmed the diagnosis. A biallelic mutation on chromosome 22 causes the disease. We have been able to demonstrate only one allelic mutation. Identification of the second is ongoing. The clinical picture of DADA2 includes systemic inflammation, immune deficiency, hematologic manifestations, and systemic vasculitis. Systemic inflammation is probably related to a proinflammatory polarization of macrophages. The inflammatory status is considered responsible for inhibiting B-cell differentiation, which sustains immunodeficiency in association with low IgM and IgG levels. Hematologic manifestations include pure red cell aplasia, although autoimmune hemolytic anemia and thrombocytopenia have been described, as well as neutropenia, the latter contributing to immunodeficiency. Systemic vasculitis affects small- and medium-sized arteries, ranging from livedo reticularis to polyarteritis nodosa. The skin and central nervous system (ischemic and hemorrhagic stroke) are mostly involved, but the kidney, liver, gastrointestinal tract, and coronary arteries can be affected too. The patient initially presented with livedo reticularis, two episodes of ischemic cerebral stroke, hyperinflammation, and aplastic and autoimmune hemolytic anemia. Before DADA2 was ruled out, she was on chronic home immunosuppressive therapy with mycophenolate and steroids to treat anemia. DADA2 diagnosis was coincident with the referral to our center for COVID-19 ARDS. The overlapping of the two diseases complicated the clinical picture, characterized by a severe hyperinflammatory condition associated with lymphopenia, hypoimmunoglobulinemia, and arterial hypertension. Table 1 summarizes the ARDS course. Treatment of inflammation and immune modulation for DADA2 and COVID-19 were initially pursued with mycophenolate, steroids (pulses included), and intravenous immunoglobulins. Anti-interleukin 1 receptor monoclonal antibody was introduced after mycophenolate discontinuation and ultimately switched to anti-tumor necrosis factor (TNF) monoclonal antibody. Both have been shown to be effective in treating COVID-19; the latter is the treatment of choice in DADA2 as a bridge to bone marrow transplantation (BMT). Thrombosis was prevented with a continuous infusion of unfractionated heparin (4, 5). During PICU stay, no source of respiratory infection was detected other than SARS-CoV-2. Immediately after admission, the patient was transitioned to helmet continuous positive airway pressure (CPAP). Prone positioning was ensured for at least 8 h/day. On PICU day 3, severe respiratory distress and high oxygen requirements (PaO_2_/FiO_2_ 150) persisted despite helmet CPAP and prone positioning, intubation was performed, and invasive MV support was started (MV settings are reported in Table 1). After initial improvement, following the institution of MV (PaO_2_/FiO_2_ 320), the ARDS worsened again (PaO_2_/FiO_2_ 129), prefigurating the need for prolonged respiratory support. This is an occurrence frequently reported in COVID-19. A percutaneous tracheostomy was carried out (modified PercuTwist technique, Rüsch, Kernen, Germany) to convey more comfort and effectiveness to long-term MV. On PICU day 25, ARDS exacerbated (PaO_2_/FiO_2_ 95) with massive ALS (Figure 1A), a common complication of COVID-19 with protracted course (2). ALS developed at unmodified MV settings, requiring bilateral chest drain placement.

**Table 1:** Clinical course of ARDS.

	**PICU day 1**	**PICU day 1**	**PICU day 3**	**PICU day 19**	**PICU day 25**	**PICU day 26**	**PICU day 26**
Events	Arrival	Admission	Transition to IMV	Percutaneous tracheostomy	Air leak	Before ECMO cannulation	On ECMO
Modality of support	HFNC	Helmet	VC-CMV	PC-CMV	PC-CMV	PC-CMV	APRV on ECMO
Settings	50 L/min FiO_2_ 0.5	CPAP 7.5 FiO_2_ 0.5	V_t_ 200 P_pl_ 28 PEEP 15 FiO_2_ 0.4 RR 16 Ti 0.9	PIP 24PEEP 12V_t_ 220FiO_2_ 0.55RR 20Ti 0.9	PIP 24 PEEP 12 V_t_ 220 FiO_2_ 0.60 RR 20 Ti 0.9	PIP 37PEEP 10V_t_ 225FiO_2_ 0.55RR 20Ti 0.9	P_high_ 25 P_low_ 5 T_high_ 2.5 T_low_ 1 V_t_ 150 FiO_2_ 0.4 RPM 3,800 BF 2.2 SG 3 FiO_2_ 1
PaO_2_/FiO_2_	190	150	320	129	120	95	175
PaCO_2_ (mmHg)	37	40	51	42	40	37	41

*HFNC, high-flow nasal cannulas; CPAP, continuous positive airway pressure (cmH_2_O); IMV, invasive mechanical ventilation; VC-CMV, volume control continuous mechanical ventilation; V_t_, tidal volume (ml); P_pl_, plateau pressure (cmH_2_O); PEEP, positive end expiratory pressure (cmH_2_O); RR, respiratory rate (breaths/minute); PC-CMV, pressure control continuous mechanical ventilation; PIP, positive inspiratory pressure (cmH_2_O); APRV, airway pressure release ventilation; P, pressure (cmH_2_O); T, time (s); RPM, revolutions per minute; BF, blood flow (L/min); SG, sweep gas (L/min)*.

Despite hyperimmune plasma administration, SARS-CoV-2 was never cleared from tracheal aspirates, probably due to the DADA2-related immunodeficiency and the need for immunosuppressive therapy. Since the patient became eligible for remdesivir administration, after previous transaminase elevation reduction, a determination was made for ECMO cannulation (PICU day 26). The assumption was that ARDS could still be potentially reversible with antiviral therapy (6). Even if the mean airway pressure was 19 cm H_2_O, the PaO_2_/FiO_2_ ratio dropped to 95. Most importantly, ALS was rapidly evolving with potential hemodynamic compromise. Thus, lung rest on extracorporeal life support appeared to be the best strategy. In this case, ECMO was taken into consideration as a last resort, given the high risk of intracranial and gastrointestinal bleeding due to DADA2 vasculitis.

To minimize the risk of viral contamination, BCDL ECMO cannulation was performed at the bedside, according to a technique previously described (3) (Figure 1). The right internal jugular vein was accessed with a 4-Fr, 5-cm hemostatic valve introducer (Terumo, Shibuya, Tokyo, Japan) after echo-guided venipuncture with a 20-gauge cannula and over the wire (0.21-in.) exchange with the introducer. Since ALS hampered TTE visualization of the heart, real-time ultrasound control was ensured with a TEE probe (S7-3t, Philips, Cambridge, MA). The linear and uncoiled progression of a 0.35-in., 260-cm-length, straight Amplatz Extra-Stiff guide wire (GW) (Cook Medical, Bloomington, IN) was followed on a bicaval mid-esophageal view (MEV): probe in mid-esophageal position with clockwise rotation, transducer angle 70–110° (7, 8). To facilitate the advancement of the GW from the superior vena cava (SVC) to the inferior vena cava (IVC), the tip was bent in a hockey stick fashion, with the same angulation as the one between the two vessels. The position of the GW and its straight and uncoiled progression were checked after each dilation on bicaval and four-chambers MEVs, excluding any looping or displacement in the right ventricle (Figures 1B,C). The absence of loops is assured if the GW generates a continuous hyperechoic image from the SVC to the IVC (Figure 1B). This is of paramount importance to prevent cardiac perforation during dilators and cannula advancement. Dilation and the final positioning of a 23-Fr Avalon Elite cannula (Maquet, Rastatt, Germany) were followed under direct visualization. TEE allowed the fine adjustment of the infusion port in front of the tricuspid valve, demonstrating the correct trajectory of the inlet jet (Figures 1D,E). Moreover, the tip-to-infusion port distance is known for each cannula diameter, i.e., 9.4 cm for the 23 Fr. Measure taking on echo and chest X-ray images allows a double check of the location of the infusion port into the right atrium (Figure 1F). The patient died on PICU day 28 because of vasculitis-related diffuse and untreatable intestinal bleeding.

## Discussion

**Figure 2:**
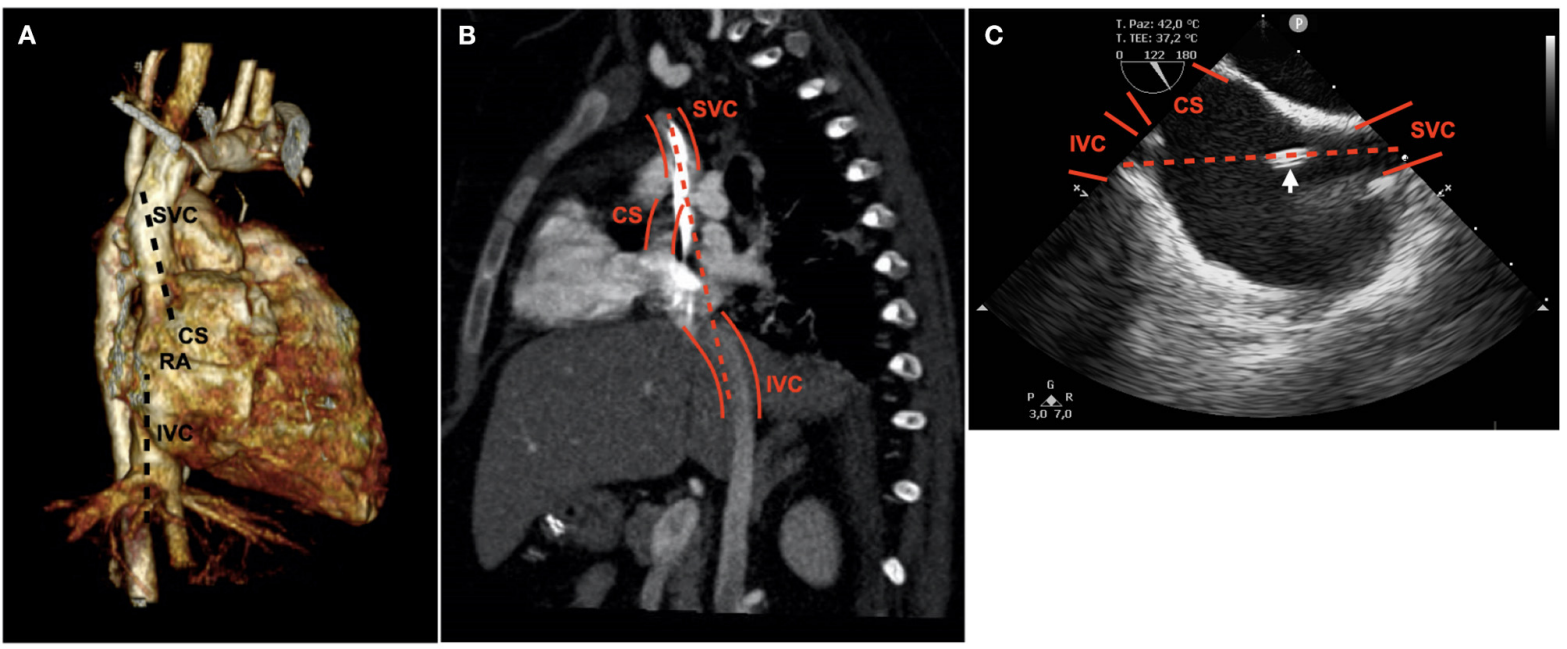
Relative position of the SVC and IVC. The IVC is more posterior and laterally displaced with respect to the SVC. RA, right atrium. The main axis of the SVC is directed toward the opening of the CS **(A,B)**. Mid-esophageal bicaval view; the CS appears misleadingly bigger than the IVC; the arrow indicates the advancing dilator. The dashed line shows the trajectory of the guidewire **(B,C)**.

Exclusively echo-guided bedside percutaneous BCDL cannulation is the approach of choice in our unit for respiratory ECMO support. The cannulation technique proved to be safe and effective with TTE guidance (3). It does not require mobilization of the patient to a fluoroscopy suite and allows expedited ECMO implementation in emergencies. This is particularly important in the case of COVID-19 patients, where infection control is a major issue. Because of the absence of an adequate TTE acoustic window due to ALS, TEE was the only option to guide cannulation. In our experience, a poor acoustic window has been recognized as a limitation of the TTE-guided cannulation approach (3). ALS, both spontaneous and associated with MV, is a frequently reported consequence of self-inflicted and ventilator-associated lung injury in COVID-19 patients (2, 6). TEE allowed an easy display of bicaval and four-chamber MEVs. Its role was pivotal in the cannulation process, effectively excluding GW and cannula displacement, and in optimization of flows and oxygenation through proper cannula positioning. In the traditional bicaval MEV at 70–110°, the coronary sinus (CS) can be misrecognized as the IVC. Simultaneous visualization of the SVC, IVC, and CS is difficult and may require additional clockwise rotation of the probe. The SVC and IVC lie on different planes, the IVC being more posterior and laterally displaced with respect to the SVC, while the SVC and CS may be easily exchanged for each other since they lie on close mid-esophageal cuts. The spatial distribution of these structures favors the concomitant visualization of the SVC and IVC in the MEV (Figure 2). Injection of microbubbles from a femoral vein in conjunction with probe manipulation are useful clues to recognize the IVC in doubtful conditions (3, 7, 8). In this regard, it is useful to recall that the flow from the IVC is deflected toward the foramen ovale by the Eustachian valve, while the bloodstream from the SVC is directed toward the TV. Thus, progression of the GW may be facilitated by blood flow toward the TV. It never happened in our experience, but when the advancement of the GW in the IVC is difficult, directional catheters like the Kumpe can be helpful in getting the IVC (9). The TEE probe that we used is suitable for pediatric patients and small adults; smaller ones are available for neonates and infants (8). It must be emphasized that the accidental progression of the GW in the CS can lead to catastrophic complications (i.e., cardiac laceration) if dilators and cannula are inadvertently advanced. In terms of patient impact and acceptability, one major strength is the avoidance of mobilization. TEE probe insertion and manipulation did not require any modifications in the administered sedation (midazolam 0.4 mg/kg/h, fentanyl 4 μg/kg/h), with no signs of discomfort. The procedure was carried out safely by three persons (cannulating intensivist, assistant, and intensivist sonographer), wearing personal protective equipment and under the spatial constraints of an isolation cubicle.

According to the ELSO Registry, children with ARDS and primary immunodeficiency have a survival rate as low as 34%. In such patients, ECMO is indicated if there is potential for long-term survival. If the patient had responded to remdesivir, she could have been bridged by anti-TNF monoclonal antibody administration to definitive treatment with BMT. Beyond 14 days of MV, ECMO survival rate drops from 56–61 to 38%. Because of the high risk of vasculitis-related bleeding, ECMO has been offered late in the course of illness (PICU day 26), when severe ALS made it the only option for survival. Of note, our patient died of untreatable intestinal bleeding (10).

## Conclusions

To our knowledge, this is the first report of exclusively TEE-guided bedside percutaneous BCDL ECMO cannulation in pediatrics. Conversely, TEE-guided BCDL cannulation is a standard approach in the adult population (11). This experience, although limited to one case, confirms our practice with TTE-guided BCDL ECMO cannulation and opens further possible perspectives in case of compromised transthoracic acoustic window. Further investigations are needed to extend the application of the described technique in pediatrics.

## Data Availability Statement

The original contributions presented in the study are included in the article/supplementary material, further inquiries can be directed to the corresponding author.

## Ethics Statement

Written informed consent was obtained from the individual(s), and minor(s)' legal guardian/next of kin, for the publication of any potentially identifiable images or data included in this article.

## Author Contributions

All authors listed have made a substantial, direct and intellectual contribution to the work, and approved it for publication.

## Conflict of Interest

The authors declare that the research was conducted in the absence of any commercial or financial relationships that could be construed as a potential conflict of interest.

## Publisher's Note

All claims expressed in this article are solely those of the authors and do not necessarily represent those of their affiliated organizations, or those of the publisher, the editors and the reviewers. Any product that may be evaluated in this article, or claim that may be made by its manufacturer, is not guaranteed or endorsed by the publisher.

## Abbreviations

PICU: pediatric intensive care unit
MV: mechanical ventilation
COVID-19: coronavirus disease 2019
SARS-CoV-2: severe acute respiratory syndrome coronavirus 2
ECMO: extracorporeal membrane oxygenation
ALS: air leak syndrome
TTE: transthoracic echo
TEE: transesophageal echo
DADA2: adenosine deaminase 2 deficiency
ARDS: acute respiratory distress syndrome
BCDL: bicaval double lumen
TNF: tumor necrosis factor
BMT: bone marrow transplantation
GW: guide wire
MEV: mid-esophageal view
IVC: inferior vena cava
SCV: superior vena cava
CS: coronary sinus
CPAP: continuous positive airway pressure.
